# Prevalence and risk factors for postpartum depression and stress among mothers of preterm and low birthweight infants admitted to a neonatal intensive care unit in Accra, Ghana

**DOI:** 10.1002/ijgo.15998

**Published:** 2024-10-30

**Authors:** John Pellegrino, Paddington T. Mundagowa, Kwame Sarfo Sakyi, Prince Gyebi Owusu, Babbel Agbinko‐Djobalar, Leila M. Larson, Mufaro Kanyangarara

**Affiliations:** ^1^ Department of Epidemiology and Biostatistics University of South Carolina, Arnold School of Public Health Columbia South Carolina USA; ^2^ Public and Environmental Wellness, School of Health Sciences Oakland University Rochester Michigan USA; ^3^ Center for Learning and Childhood Development‐Ghana Accra Ghana; ^4^ Michigan State University East Lansing Michigan USA; ^5^ Korle‐Bu Teaching Hospital Accra Ghana; ^6^ Department of Health Promotion Education and Behavior University of South Carolina, Arnold School of Public Health Columbia South Carolina USA

**Keywords:** low birth weight, maternal mental health, neonatal intensive care unit, perceived stress, postpartum depression, preterm

## Abstract

To determine the prevalence of postpartum depression (PPD) and postpartum stress (PPS) and identify associated risk factors among mothers of preterm and low birth weight (LBW) infants. We conducted a secondary analysis of data collected from 255 mothers with preterm and LBW infants admitted to the neonatal intensive care unit (NICU) at Korle‐Bu Teaching Hospital, Accra, Ghana. A standardized interviewer‐administered questionnaire collected data on maternal, pregnancy, birth, and infant characteristics. The questionnaire also included the Patient Health Questionnaire‐9 (PHQ‐9) and the Perceived Stress Scale‐4 (PSS‐4) to assess PPD and PPS, respectively. Simple and multivariable linear regression analyses were performed to identify factors associated with PPD and PPS. The prevalence of moderate to moderately severe PPD was 3.9%, and that of PPS was 43.5%. The multivariable linear regression analysis showed that an increased number of prenatal care visits (*β*‐estimate = 0.26; 95% confidence interval [CI] 0.08–0.43; *P* < 0.01) was positively associated with higher scores on the PHQ‐9, whereas gestational age at birth (*β* = −0.21; 95% CI –0.40 to −0.03; *P* = 0.02) was inversely associated with PHQ‐9 scores. Moreover, a longer gestational period at the first prenatal care visit (*β* = 0.25; 95% CI 0.05–0.45; *P* = 0.01) and following the Islamic religion were associated with elevated scores on the PSS‐4 (*β* = 0.95; 95% CI 0.11−1.80; *P* = 0.011). Our findings underscore the presence of moderate PPD levels and high PPS levels among mothers. Active screening, diagnosis, and treatment for mothers at risk of mental health disorders during the peripartum period could enhance coping mechanisms for mothers navigating the challenging NICU environment and transitioning to the home environment.

## Synopsis

The study revealed relatively low postpartum depression but high postpartum stress among mothers of preterm and low birth weight infants at a hospital in Ghana.

## 1 INTRODUCTION

Maternal mental health disorders constitute a significant cause of disability in the postpartum period. Postpartum depression (PPD) and postpartum stress (PPS) are among the most common maternal mental health conditions, yet they are frequently underdiagnosed, untreated, and underreported.^1^ PPD is defined as a major depressive episode within 4 weeks post‐childbirth, with symptoms ranging from sadness, anxiety, fatigue, despair and mood changes to self‐harm or suicidal thoughts.^2^ PPS is defined as a distinct negative emotional state produced by postpartum stressors.^3^ Although stress is a normal response to challenging situations, high levels of stress may be associated with functional impairment and poor maternal and infant outcomes.^4^ The burden of PPD and PPS is particularly pronounced in low‐ and middle‐income countries (LMICs) compared with high‐income countries.^5^ In LMICs, the prevalence of perinatal mental health disorders during pregnancy is 15.6%, rising to 19.8% following childbirth.^6^

Numerous risk factors contribute to the higher prevalence of perinatal mental health disorders in LMICs, including low socioeconomic status, limited education, lack of employment opportunities, domestic abuse, age, parity, history of anxiety or depression, and insufficient psychosocial support.^7^, ^8^, ^9^ Additionally, the integration of mental health services with maternal health services is lacking in many LMICs,^10^ resulting in missed opportunities for diagnosis and treatment. A persistent shortage of mental health professionals, inadequate access to support, cultural barriers to seeking help, and unresolved socioemotional and financial difficulties perpetuate these disorders.^5^, ^7^, ^8^, ^9^

Mothers of preterm and low birth weight (LBW) infants face nearly double the risk of PPD compared with mothers of full‐term infants.^11^, ^12^ The prevalence of PPD and PPS among mothers of preterm and LBW infants admitted to neonatal intensive care units (NICUs) ranges between 29%–40%^11^ and 23%–35%,^13^ respectively. Extended NICU stays exacerbate stress, with some NICUs separating mothers from their infants and limiting the opportunity for early bonding.^14^

Maternal mental health symptoms are most intense during the initial 6 weeks post‐delivery and may persist after hospital discharge because of uncertainty about the child's prognosis, social stigma and the increased caregiving demands for a preterm and LBW infant.^11^, ^12^, ^13^ Mothers of preterm and LBW infants often experience overwhelming feelings of sadness, guilt, fear, anger, and low self‐esteem, which can lead to a loss of interest in activities, hopelessness, disrupted sleep, and suicidal thoughts.^11^, ^12^, ^13^ Maternal mental health disorders also have implications for child development and the overall family wellbeing, especially when the condition becomes chronic.^15^ PPD is associated with reduced odds of breastfeeding, infant neglect, poor sleep, and sociopathic behavior.^16^ Children of mothers with PPD are more likely to cry excessively, develop diarrhea and other infectious diseases, and internalize problems that precede depression and anxiety later in life.^17^ Consequently, early screening and diagnosis of maternal mental health disorders are crucial for informing interventions to improve maternal and child health, particularly in LMICs such as Ghana.

Ghana has a high prevalence of PPD, ranging from 4% to 41%.^18^, ^19^, ^20^ NICU admission triggers significant PPS because of the intense worry about the infant's health, the emotional strain of separation, the challenges of navigating complex medical decisions, the disruption of family expectations and routines, and accumulating hospital costs.^21^ However, there remains a dearth of data on maternal mental health disorders and their associated risk factors among mothers of preterm and LBW infants. This study aims to determine the prevalence and risk factors of PPD and PPS among mothers of preterm and LBW infants in Ghana. The findings of this study will be critical in addressing the information gaps regarding maternal mental health and guiding the development of evidence‐based interventions in Ghana.

## 2 MATERIALS AND METHODS

### 2.1 Study setting and population

This study was a secondary analysis of data collected as part of a validation study to determine the accuracy of the BEMPU® TempWatch in detecting neonatal hypothermia among preterm and LBW infants admitted to the NICU at Korle‐Bu Teaching Hospital in Accra, Ghana. This hospital is the largest health referral center in West Africa with a capacity of 2000 beds. The hospital NICU caters to the health needs of preterm and critically ill infants, with an annual average of 2000 admissions. The NICU has a capacity of 60 cots, incubators, and warming platforms, but occupancy frequently exceeds its designated capacity. Infants weighing under 2000 g are monitored in incubators, and those over 2000 g are wrapped and nursed in cots. The current model of care requires separation of the infant from their family, with limited opportunities for bonding due to the clinical environment and space limitations. An additional room is designated for kangaroo mother care and accommodates up to six mother‐infant pairs at a time.

For the validation study, mother‐infant pairs were eligible to participate if the infant was admitted to the NICU, aged less than 28 days old, was preterm or LBW, and demonstrated clinical stability. Participants were excluded from the study if the mother was younger than 18 years of age or expected to be discharged within 24 h. Study nurses identified eligible infants, provided an overview of the study, and sought maternal written informed consent before enrollment. Sample size calculation was based on a one‐sample equivalence test, anticipating sensitivity of 90% for the BEMPU® TempWatch, statistical power of 80%, an estimated 15% loss to follow up, 15% threshold for equivalence, and a type‐I error rate of 5%. A sample size of 255 was deemed sufficient for ensuring statistical validity.

### 2.2 Study procedures

Trained study nurses administered a pretested standardized questionnaire to mothers. Pretesting phase data were not included in the analysis. The questionnaire collected information on sociodemographic characteristics, medical history, perinatal health, anthropometric measurements of the infant and maternal symptoms of depression and stress. Assessment of PPD and PPS was conducted within the first 4 weeks postpartum using the Patient Health Questionnaire‐9 (PHQ‐9)^22^ and the Perceived Stress Scale‐4 (PSS‐4),^23^ respectively.

The PHQ‐9 scale is a commonly used screening tool for assessing varying levels of depression.^22^ It consists of nine questions probing the frequency of depressive symptoms in the preceding 2 weeks. Responses were measured on a four‐point Likert scale: 0 (not at all), 1 (several days), 2 (more than half the days), and 3 (nearly every day). Based on the total score, ranging from 0 to 27, depression severity can be classified into minimal (0–4), mild (5–9), moderate (10–14), moderately severe (15–19), and severe (20–27) categories. Although the Edinburgh Postnatal Depression Scale is commonly used for postpartum depression, the PHQ‐9 was chosen based on its accessibility, ease of use, and validation and previous use among postpartum African women.^19^, ^20^, ^24^

The PSS‐4 scale is designed to gauge perceived stress by inquiring about feelings and thoughts during the preceding month.^23^ This scale comprises four questions focusing on personal control, confidence, and difficulties. Responses are ranked on a five‐point Likert scale ranging from 0 (never) to 4 (very often). A cumulative score of at least 6 indicates elevated levels of perceived stress. In this study, mothers with a PHQ‐9 score of 10 or more and a PSS‐4 scale of 6 or more were subsequently referred to a primary care counselor for mental health counseling services, as these scores are considered clinically significant indicators of depression and stress. The PSS‐4 scale has been used in several studies in Ghana,^25^, ^26^, ^27^ but, to our knowledge, it has not been validated in a population similar to our sample.

Data were captured and securely stored on password‐protected Android tablets using Open Data Kit (ODK) software. To minimize data entry errors and missingness, field validation checks were incorporated into ODK. Data were collected from May 2021 to January 2022 and ethical approval for human‐subject research was obtained from the Institutional Review Boards at both the University of South Carolina (Protocol 00095600) and Korle‐Bu Teaching Hospital (KBTH‐IRB/00052/2020).

### 2.3 Statistical analysis

The primary outcomes of this study were PPD and PPS. Descriptive statistics were conducted, with continuous variables presented as means and standard deviations. The *t* test was used to compare means between individuals with or without PPD or PPS. Continuous variables were tested for normality and considered not normally distributed if the Shapiro–Wilk test *P* value was less than 0.05, in which case, the Mann–Whitney *U* test *P* value was reported. Simple linear regression analyses were conducted and all variables with *P* values below 0.2 were added into the multivariable model in which statistical significance was set at *P* < 0.05. Multicollinearity was assessed by calculating the variance inflation factor; independent variables with high variance inflation factors (≥4) were dropped. All statistical analyses were performed using SAS 9.4.

## 3 RESULTS

A total of 255 mother‐infant pairs participated in this study. Of these, 205 (80%) were married or living with a partner, 230 (90%) had attained at least a middle school education, 228 (89%) practiced Christianity, 130 (51%) identified as Akan ethnicity, 179 (70%) were employed, and 136 (53%) delivered via cesarean section (Table 1). The mean maternal age was 29.9 ± 6.4 years, the mean number of prenatal care (PNC) visits was 6.6 ± 2.4, and the mean parity was 2.0 ± 1.3. Most infants were female (148, 58%) and part of a singleton birth (193, 76%), with a mean gestational age at birth of 32.1 ± 3.1 weeks, and a mean birth weight of 1542.0 ± 439.8 g.

**TABLE 1:** Characteristics of study participants overall and by depression and stress status (*n* = 255).^a^

Characteristics	
Maternal characteristics	
Maternal age, years	29.9 ± 6.4
Level of education	
Primary education or less	25 (10)
Middle school or higher	230 (90)
Marital status	
Never married	50 (20)
Married or living together	205 (80)
Employment status	
Employed	179 (70)
Unemployed	76 (30)
Religion	
Christian	228 (89)
Islam	27 (11)
Ethnicity	
Akan	130 (51)
Other	131 (49)
Number of household members^b^	
≤2	101 (44)
>2	126 (56)
Birth and pregnancy	
Number of pregnancies	2.6 ± 1.7
Parity	2.0 ± 1.3
Prenatal care visits^c^	6.6 ± 2.4
Mode of delivery	
Vaginal	119 (47)
Cesarean section	136 (53)
Type of birth	
Singleton	193 (76)
Multiple	62 (24)
Infant characteristics	
Sex	
Male	107 (42)
Female	148 (58)
Gestational age at birth, weeks	32.1 ± 3.1
Birth weight, g	1542.0 ± 439.8
Psychometric scores	
PHQ‐9 score	2.7 ± 3.4
PSS‐4 score	5.1 ± 2.5

Abbreviations: PHQ‐9, Patient Health Questionaire‐9; PSS‐4, Perceived Stress Scale‐4.

^a^ Data are presented as mean ± standard deviation or as number (percentage).

^b^ 28 missing.

^c^ 37 missing.

The mean score on the PHQ‐9 was 2.7 ± 3.4. Among the interviewed mothers, 193 (76%) experienced minimal PPD, 51 (20%) experienced mild PPD, 6 (2%) experienced moderate PPD, and 4 (2%) experienced moderately severe PPD. Importantly, none of the mothers scored above 20 on the PHQ‐9 scale, a threshold indicative of severe depression. Additionally, the mean PSS‐4 score was 5.1 ± 2.5, with 111 (44%) participants with scores above the cut‐off of 6 indicating PPS.

The results of the simple and multivariable linear regression analyses examining the associations with PPD and PPS are presented in Tables 2 and 3, respectively. In the simple linear regression analysis, the number of PNC visits emerged as a factor significantly associated with PPD (*β* = 0.22; 95% CI 0.05–0.09; *P* = 0.014) (Table 2). The multivariable analysis revealed that, on average, a one‐unit increase in the number of PNC visits was associated with a 0.26 (*β* = 0.26; 95% CI 0.08–0.43; *P* = 0.004) unit increase in the PPD score, assuming the other predictor variables were held constant. Additionally, for every additional week of gestational age, there was a 0.21‐unit reduction in the PPD score (*β* = −0.21; 95% CI: −0.40 to 0.03; *P* = 0.022).

**TABLE 2:** Simple and multivariable linear regression analysis of the PHQ‐9 scores (*n* = 255).

Variable	Simple linear regression		Multivariable linear regression	
	Beta (95% CI)	*P* value	Beta (95% CI)	*P* value
Maternal age, years	−0.04 (−0.11 to 0.02)	0.287		
Never married vs. married or living with a partner	0.02 (−1.04 to 1.08)	0.968		
Primary education or less vs. middle school or higher	−0.65 (−2.06 to 0.76)	0.367		
Islam vs. Christian	−0.70 (−2.06 to 0.66)	0.312		
Other vs. Akan	<0.01 (−0.01 to 0.01)	0.942		
Unemployed vs. employed	−0.34 (−1.24 to 0.58)	0.468		
Number of household members	0.02 (−0.14 to 0.18)	0.793		
Number of pregnancies	0.08 (−0.17 to 0.33)	0.518	–	–
Parity	−0.10 (−0.42 to 0.21)	0.518		
Multiple vs. singleton birth	−0.38 (−1.36 to 0.60)	0.448		
Cesarean section vs. vaginal delivery	0.28 (−0.56 to 1.12)	0.513		
Number of PNC visits	0.22 (0.05–0.39)	0.014	0.26 (0.08–0.43)	0.004
Gestational month of the first PNC visit	−0.03 (−0.40 to 0.30)	0.868		
Gestational age at birth, wk	−0.09 (−0.23 to 0.07)	0.108	−0.21 (−0.40 to −0.03)	0.022
Male vs. female	−0.15 (−1.00 to 0.70)	0.734		
Birth weight, g/100	−0.08 (−0.20 to 0.01)	0.081	0.02 (−0.11, 0.15)	0.724

Abbreviations: CI, confidence interval; PNC, prenatal care.

**TABLE 3:** Simple and multivariable linear regression analysis of the PSS‐4 scores (*n* = 255).

Variable	Simple linear regression		Multivariable linear regression	
	Beta (95% CI)	*P* value	Beta (95% CI)	*P* value
Maternal age, years	<−0.01 (−0.04 to −0.04)	0.990		
Never married vs. married or living with a partner	0.40 (−0.21 to 1.02)	0.203		
Primary education or less vs. middle school or higher	−0.29 (−1.11 to 0.53)	0.482		
Islam vs. Christian	0.74 (−0.04 to 1.53)	0.061	0.95 (0.11–1.80)	0.027
Other vs. Akan	<−0.01 (−0.01 to <0.01)	0.603		
Unemployed vs. employed	0.51 (−0.03 to 1.04)	0.058	0.14 (−0.44 to 0.71)	0.645
Number of household members	−0.05 (−0.14 to 0.04)	0.260		
Parity	−0.05 (−0.23 to 0.14)	0.604		
Multiple vs. singleton birth	−0.19 (−0.76 to 0.38)	0.511		
Cesarean section vs. vaginal delivery	−0.14 (−0.63 to 0.35)	0.560		
Number of PNC visits	0.03 (−0.08 to 0.14)	0.624		
Gestational month of the first PNC visit	0.23 (0.03–0.43)	0.020	0.25 (0.05–0.45)	0.011
Gestational age at birth, wk	−0.01 (−0.09 to 0.07)	0.823		
Male vs. female	0.05 (−0.56 to 0.67)	0.869		
Birth weight, g/100	0.02 (−0.05 to 0.09)	0.604		

Abbreviations: CI, confidence interval; PNC, prenatal care.

In terms of PPS, the simple linear regression indicated that only the gestational age at the first PNC visit was significantly associated with PPS (*β* = 0.23; 95% CI 0.03–0.43; *P* = 0.020) (Table 3). The multivariable linear regression model showed a 0.95 unit increase in PSS‐4 score (*β* = 0.95; 95% CI 0.11–1.80; *P* = 0.027) among mothers who identified with Islam compared with mothers who identified with Christianity. Also, on average, each additional gestational month for the first PNC visit was associated with a 0.25‐unit increase in PSS‐4 scores (*β* = 0.25; 95% CI 0.05–0.45; *P* = 0.011). Variance inflation factors for the independent variables included in both the PPD and PPS models were all below 2.0, indicating the absence of multicollinearity.

## 4 DISCUSSION

The study aimed to determine the prevalence of PPD and PPS among mothers of preterm and LBW infants in Ghana and to identify associated maternal, neonatal, and obstetric factors. The findings reveal a substantial burden of maternal mental health disorders, with 3.9% of mothers experiencing moderate to moderately severe depression and 43.5% reporting elevated levels of stress. Factors significantly associated with higher PHQ‐9 scores included the number of PNC visits, and gestational age at birth, while maternal religion and gestational month of the first PNC visit were identified as risk factors for PPS. These results underscore the urgent need for mental health interventions, particularly for mothers of preterm and LBW infants.

The prevalence of moderate to moderately severe PPD (3.9%) observed in our study was on the lower end of the range of estimates reported in other studies in Ghana (4%–41%).^18^, ^19^, ^20^ This difference could be attributed to variations in screening tools; while our study used the PHQ‐9, other studies used the Edinburgh Postnatal Depression Scale,^18^, ^19^, ^28^ which is known to yield higher prevalence rates because of its greater sensitivity in detecting depressive symptoms during the postpartum period.^29^ Additionally, some previous studies sampled a predominantly rural population or were community‐based not facility‐based.^19^

Although no direct comparisons within the Ghanaian context exist, our study found a high prevalence of PPS (43.5%). The concept of PPS is not a well‐established clinical entity, particularly from a psychiatric perspective. However, in this study, PPS was defined as the perceived stress experienced by mothers in the postpartum period. Although the clinical significance of PPS is not fully understood, providing psychosocial support to reduce stress may improve both maternal well‐being and infant outcomes. Adopting family‐centered care practices in the NICU could provide emotional support, reduce maternal stress, enhance bonding between parents and their newborns, and facilitate better communication between healthcare providers and families.^30^ Particularly in settings with limited mental health resources, such a model can help alleviate the psychological burden on mothers by addressing not only the clinical needs of the infant but also the emotional and psychosocial needs of the family.

An interesting and somewhat unexpected finding in our study, that warrants further investigation, was the positive association between the number of PNC visits and higher PPD scores. PNC is generally thought to enhance maternal self‐efficacy and reduce the risk of mental health issues; our results suggest a more complex relationship. One possible explanation is that mothers who attend more PNC visits may do so due to complications during pregnancy,^31^ which could heighten anxiety and increase the likelihood of PPD. This hypothesis aligns with previous studies that have identified pregnancy complications as a risk factor for PPD.^11^, ^32^ On the other hand, a recent study in Ethiopia found that prenatal depression negatively impacted PNC service utilization, suggesting that maternal mental health issues may influence the perceived benefit of PNC services.^33^ Although our findings diverge from studies where higher PNC use is associated with reduced PPD risk,^34^ this discrepancy may also reflect differences in the quality of PNC services across settings,^35^ suggesting that merely increasing the number of PNC visits may not be sufficient to mitigate the risk of PPD and PPS.

Relatedly, our study found an association between later gestational age at the first PNC visit and increased PPS levels, which may also reflect the compounding stress of managing a high‐risk pregnancy. This finding is concerning, given that over half of women in sub‐Saharan Africa delay their first PNC visit beyond the recommended timeframe.^36^ Timely initiation of PNC is essential for early identification and management of pregnancy‐related complications such as preterm birth and LBW, which are known to increase maternal stress. Our findings suggest that mothers who delay their first PNC visit may face additional psychological burdens, possibly as a result of the compounding stress of managing a high‐risk pregnancy. Addressing the barriers to early and sustained PNC engagement is crucial, as timely PNC can help reduce both PPD and PPS risks. Furthermore, incorporating mental health screening and interventions into PNC services could be particularly beneficial for mothers at risk of delivering preterm or LBW infants.

Our study highlights the significance of gestational age as a protective factor against PPD. For each additional week of gestation, the likelihood of experiencing PPD decreased, which is consistent with previous research showing that mothers of infants with greater prematurity are at higher risk of PPD.^11^ Extremely and very preterm or LBW infants are often fragile, with a higher mortality risk, which can be emotionally traumatic to the mother. The uncertainty associated with poor prognosis and the high neonatal mortality risk can contribute to maternal distress. Furthermore, mothers of preterm and LBW infants may encounter social stigma due to the physical appearance of their newborn, as many people are unfamiliar with infant size.^37^

Another significant finding from our study is the association between maternal religion and PPS, with Islamic mothers experiencing higher stress levels than Christian mothers. Similar findings were also reported in a study in the United Arab Emirates, where Islamic mothers also reported higher levels of stress.^38^ Cultural practices, such as the Islamic tradition of staying home for 40 days postpartum,^39^ may be disrupted by the NICU environment, potentially increasing stress. However, it is important to interpret these findings with caution, given the underrepresentation of Islamic mothers in our study sample (10.6%). Nevertheless, religion plays a major role in offering spiritual and social support for postpartum mental health.^40^, ^41^ Spiritual care has been shown to augment coping mechanisms, reduce anxiety, and foster mother‐infant interactions.^41^ The findings underscore the need to recognize the role of religion in postpartum mental health and engage with faith‐based organizations in designing and implementing interventions to address maternal mental health needs in diverse cultural contexts.

Our study has several limitations. The cross‐sectional study design precludes establishing causal relationships between the identified risk factors and PPD or PPS. Additionally, self‐reported symptoms of depression and stress are susceptible to social desirability bias. We did not assess history of mental health symptoms, which could have impacted the prevalence of PPD and PSS in our study. The study was limited to a single hospital, which may affect the generalizability of our findings. Nevertheless, as a large referral hospital, Korle‐Bu Teaching Hospital draws patients from a broad catchment area. We also acknowledge that the prevalence of PPD and PPS reported in this study may be an overestimation because the present study included mothers within the first 4 weeks of childbirth, a period known for intense hormonal fluctuations, fatigue, and mood swings, also known as the “baby blues.” Additionally, the study coincided with the peak of the COVID‐19 pandemic, which may have amplified stress and depression levels due to pandemic‐related stressors.

In conclusion, the present study underscores the presence of moderate levels of depression and high levels of perceived stress among mothers of preterm and LBW infants in Ghana. Identified risk factors include PNC attendance, young maternal age, gestational age at birth, and maternal religion. Healthcare providers should implement screening protocols and enhance coping strategies addressing the unique needs of mothers of infants in the NICU. Prioritizing maternal mental health will also enhance child growth, development, and survival.

## AUTHOR CONTRIBUTIONS

J.P. and P.T.M. performed the data analysis and drafted the manuscript. M.K. conceived the study design, acquired the funding, coordinated the study, performed the data analysis, and drafted the manuscript. K.S.S., P.G.O., and B.A.D. coordinated the study, collected the data, and provided critical revisions to the manuscript. L.M.L. provided guidance on the data analysis and critical revisions to the manuscript. All authors read and approved the final manuscript.

## FUNDING INFORMATION

This work was supported by the Thrasher Research Fund (award number 15194).

## CONFLICT OF INTEREST STATEMENT

The authors have no conflict of interest.

## Data Availability

The data that support the findings of this study are available from the corresponding author upon reasonable request.
